# The Chemical Composition of Essential Oils from *Cinnamomum camphora* and Their Insecticidal Activity against the Stored Product Pests

**DOI:** 10.3390/ijms17111836

**Published:** 2016-11-04

**Authors:** Shanshan Guo, Zhufeng Geng, Wenjuan Zhang, Junyu Liang, Chengfang Wang, Zhiwei Deng, Shushan Du

**Affiliations:** 1 Beijing Key Laboratory of Traditional Chinese Medicine Protection and Utilization, Beijing Normal University, Beijing 100875, China; guoshanshan@mail.bnu.edu.cn (S.G.); gengzhufeng@bnu.edu.cn (Z.G.); zwj0729@mail.bnu.edu.cn (W.Z.); liangjunyu@nwnu.edu.cn (J.L.); dengzw@bnu.edu.cn (Z.D.); 2 Analytical and Testing Center, Beijing Normal University, Beijing 100875, China

**Keywords:** *Cinnamomum camphora*, *Tribolium castaneum*, *Lasioderma serricorne*, fumigant activity, contact activity

## Abstract

To investigate the chemical composition and insecticidal activity of the essential oils of certain Chinese medicinal herbs and spices, the essential oils were extracted from the stem barks, leaves, and fruits of *Cinnamomum camphora* (L.) Presl, which were found to possess strong fumigant toxicity against *Tribolium castaneum* and *Lasioderma serricorne* adults. The essential oils of the plants were extracted by the method of steam distillation using a Clavenger apparatus. Their composition was determined by gas chromatography/mass spectrometric (GC-MS) analyses (HP-5MS column), and their insecticidal activity was measured by seal-spaced fumigation. D-camphor (51.3%), 1,8-cineole (4.3%), and α-terpineol (3.8%), while D-camphor (28.1%), linalool (22.9%), and 1,8-cineole (5.3%) were the main constituents of its fruits. The essential oils of the *C. camphora* all showed fumigant and contact toxicity. Other compounds exhibited various levels of bioactivities. The results indicate that the essential oils of *C. camphora* and its individual compounds can be considered a natural resource for the two stored-product insect management.

## 1. Introduction

The red flour beetle (*Tribolium castaneum* Herbst) and the cigarette beetle (*Lasioderma serricorne* Fabricius) are worldwide pests of stored products. They are dominant populations in the stored traditional Chinese medicines insect community [1]. The principal method to control these insects is to use synthetic insecticides or fumigants, but that may cause health hazards to warm-blooded animals, pose risk of environmental pollution, and bring development of resistance by insects or pest resurgence [2]. Therefore, an increasing number of scientists have been keen on searching for active natural products to use as botanical insecticides [3,4]. Essential oils continue to be a subject of interest among the international research community, which include lipids, terpenoids, ketones, phenols, and oxygenated derivatives and have been found for their control effects [4,5,6,7,8,9].

During the screening process for new agrochemicals from Chinese medicinal materials and spices, the essential oils of *Cinnamomum camphora* (L.) Presl (commonly known as camphor tree, camphorwood or camphor laurel) were found to possess fumigant and contact activities against the red floor beetle and the cigarette beetle. *C. camphora* is a tree native to South China, Taiwan, South Japan, Korea, and Vietnam [10]. It is cultivated as an ornamental plant and as a good material for wood furniture. Additionally, as a source of camphor and camphor oil, this plant is an important source for perfume. In the medical field, this plant can be used to treat muscular strains, inflammation, and rheumatic conditions [11].

In previous research, *C. camphora* extracts have shown many kinds of bioactivities against *T. castaneum*, such as the contact activity [12,13], the fumigant activity [14], the repellent activity [12,14], as well as progeny suppression [13,14]. However, those studies only tested the bioactivities of powders/essential oils from *C. camphora* aerial parts and seldom mentioned the bioactive compounds from them. Moreover, although some studies have analyzed the chemical composition of the different parts from essential oils [15,16,17,18], very little work has been done on the stem barks of this plant. Some species of the *Cinnamomum* genus have been confirmed to have insecticidal activities on their barks [19,20], whereas there has been no report on the bioactivity of *C. camphora*. Particularly, we have already analyzed the essential oil of *C. camphora* leaves (EL, vegetative organs of this plant) and found it to possess strong bioactivities against *L. serricorne* in our former study [18]. At this time, we bring our attention to its protective and reproductive organs, namely barks and fruits, and we try to find other bioactive compounds from its essential oils as well.

Thus, in this work, we analyzed the chemical composition of the essential oils of stem barks (EB) for the first time and compared the chemical composition between EB, EL, and the essential oils of its fruits (EF). In addition, we investigated the fumigant and contact activities of EB, EL, and EF against *T. castaneum* and *L. serricorne* adults, and found bioactive compounds from its essential oils.

## 2. Results

### 2.1. Chemical Composition of Essential Oils

**Figure A1:**
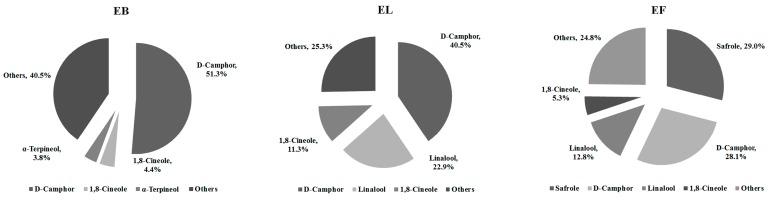
The major components of essential oils.

The gas chromatography/mass spectrometric (GC-MS) analysis results for the *C. camphora* essential oils are summarized in Table 1 and Figure A1. The yields of EB, EL, and EF were 0.42%, 1.83%, and 1.18% (*v*/*w*), respectively. The main components of EB were D-camphor (51.3%), 1,8-cineole (4.3%), α-terpineol (3.8%), and 3-methyl-2-butenoic acid, oct-3-en-2-yl ester (3.1%), while safrole (29.0%), D-camphor (28.1%), linalool (12.8%), and 1,8-cineole (5.3%) were the main constituents of EF. Twenty-seven components were identified in EB, while only 17 constituents identified in EF. Some compounds are unique in EB, such as γ-terpinen, isoterpinolene, 1,3,8-*p*-menthatriene, terpinen-4-ol, α-terpineol, eugenol, β-cadinene, and α-cubebene.

### 2.2. Insecticidal Activity

The results of fumigant assays for the essential oils against *T*. *castaneum* and *L. serricorne* adults are presented in Table 2. EB and EL exhibited strong fumigant toxicity with LC_50_ values both less than 3.2 mg/L air; meanwhile, EF also showed strong fumigant toxicity against the red flour beetle (LC_50_ = 8.5 mg/L air). Moreover, the essential oils showed contact activity against the two stored-product insects, and the results are presented in Table 3. EB and EL were not so effective against *T*. *castaneum* in our contact toxicity measure range, and their LD_50_ values could not be calculated (LD_50_ > 50.0 µg/adult); however, they exhibited contact toxicity against *L. serricorne* adults with LD_50_ values of 7.6 and 21.3 µg/adult, respectively. EF showed contact toxicity against *T*. *castaneum* and *L. serricorne* adults with LD_50_ values of 19.0 and 10.1 µg/adult, respectively.

The bioactivities of five major individual compounds (D-camphor, linalool, 1,8-cineole, safrole, and α-terpineol) were tested, and the results are listed in Table 2 and Table 3. As the most abundant constituents in EB (51.3%) and EL (40.5%), D-camphor showed strongest fumigant activity (LC_50_ < 2.3 and LC_50_ = 2.4 mg/L air, respectively) against *T*. *castaneum* and *L. serricorne* adults. It is the main contributor to the fumigant activity of EB and EL. However, D-camphor (LD_50_ > 50.0 µg/adult) may also have led to the low contact activity of EB and EL against *T*. *castaneum* adults. Furthermore, the five major individual compounds showed similar contact activity (LD_50_ = 13.4, 12.7, 14.6, 15.6, and 12.0 µg/adult) with EB, EL, and EF (LD_50_ = 7.6, 21.3, and 10.1 µg/adult) against *L. serricorne* adults.

Because we failed to test the bioactivities of safrole, we only compared the fumigant and contact results with the study with similar conditions [21,24]. As the uppermost constituent (29.0%) in EF, safrole showed weak fumigant activity against *T*. *castaneum* adults with a LC_50_ value of 38.3 mg/L air. However, it had the strongest contact activity (LD_50_ = 4.7 µg/adult) among the tested individual compounds against *T*. *castaneum* adults. Because EF had fumigant activity with LC_50_ values of 8.5 mg/L air, it was not safrole but D-camphor and 1,8-cineole (LC_50_ < 2.3 mg/L air and LC_50_ = 5.5 mg/L air, respectively) that were the main contributors to its fumigant activity against *T*. *castaneum* adults.

## 3. Discussion

There have not been any reports about EB of *C. camphora*, but some researchers have identified compounds in EF from different areas. The essential oil of fruits cultivated in the province of Guizhou contained D-camphor (26.1%), 1,8-cineole (19.9%), linalool (9.2%), α-terpineol (7.2%), and limonene (5.3%) [15]. The main constituents in the sample from Jiangxi were D-camphor (42.8%), 1,8-cineole (24.8%), α-terpineol (8.7%), and β-pinene (5.8%) [16]. Compared with our previous study on EL (Table 1), the EB, EL, and EF all had a large percentage of D-camphor (28.1%–51.3%) and a certain percentage of 1,8-cineole (4.4%–11.3%). EL and EF both contained linalool (12.8%–22.9%), while safrole was retrieved only in EF. The prior paper also studied the chemical composition of the twigs and seeds of *C. camphora* in Jiangxi [25]. The main compounds of this essential oil were eucalyptol (17.2%), camphor (13.2%), and 3,7-dimethyl-1,3,7-octatriene (11.5%), while in seeds they were eucalyptol (20.9%), methyleugenol (20.0%), linalool (14.7%), and camphor (5.5%). The content and distribution of the essential oils constituents were influenced by tissue and organ diversity and metabolic pathways in the plant.

In previous works, powders and other parts of *C. camphora* essential oils have been evaluated for bioactivities against agricultural as well as stored product insects [12,13,14,17,18,25,26,27,28,29,30,31,32,33,34,35,36,37,38,39]. The selected constituents also showed bioactivities and reflected the compositional complexity in bioactivity of natural mixtures [40]. Besides these main constituents, plenty of minor components are contained in essential oils as well. The synergistic effects of these major and minor components jointly determined the bioactivity properties of essential oils. These defense systems of plants usually worked together, not as single ones. Minor constituents also enhanced the insecticidal effectiveness of the major ones [41].

On the other hand, some constituents of the essential oils of *C. camphora* still need safety risk assessment. As the uppermost constituent in EB and EL, the daily maximum human therapeutic dose of D-camphor is about 1.43 mg/kg. This dose is relatively safe, but still lacks information of long-term experiments [42]. Although safrole accounting for 29.0% of the EF showed obvious insecticidal activity, it is carcinogenic. Linalool, based on the volume of use (2011) from the International Fragrance Association, harmed the aquatic compartment [43,44]. Moreover, at very low doses, 1,8-cineole can be used as a flavoring and medicinal ingredient; however, in higher-than-normal doses, it is hazardous for behavior, the respiratory tract, and the nervous system [45]. Although the biological effects of the individual components of essential oils are known, the toxicokinetics and ecotoxicology of their blends and single compounds is still much more difficult to evaluate [20,46]. In general, medicinal herbs are low-risk and relatively well studied experimentally and clinically. However, no experimental data about the safety of the essential oils of the barks leaves and fruits are yet available. Further study should be focused on not only the effectiveness but also the safety of essential oils in order to manage these insects.

## 4. Materials and Methods

### 4.1. Insects

*T. castaneum* and *L. serricorne* were reared on wheat flour mixed with yeast (10:1, *w*/*w*) in dark incubators at 29 ± 1 °C and 70%–80% relative humidity. Adult insects of mixed sex, about 1 week old, were used for bioassay tests.

### 4.2. Plant Materials and Essential Oils Extraction

Stem barks, leaves, and fruits of *C. camphora* were all collected in May 2013 from Suzhou City (31.97° N and 120.49° E), Jiangsu, China. The samples were identified as *Cinnamomum camphora* (L.) Presl. The voucher specimens (BNU-dushushan-2013-05-25-005, BNU-dushushan-2013-05-25-006, and BNU-dushushan-2013-05-25-007) were deposited at the Herbarium (BNU) of College of Resources Science and Technology, Beijing Normal University. In this work, essential oils were extracted from barks and fruits (1 kg and 0.745 kg, respectively, of dry matter) with hydrodistillation. A modified Clevenger-type apparatus was used, and each extraction process lasted 6 hours. The essential oils were dehydrated with anhydrous sodium sulfate and stored in airtight containers in a refrigerator at 4 °C.

### 4.3. Chemical Components Determination

GC-MS analysis was run on an Agilent 6890 N gas chromatograph connected to an Agilent 5973 N mass selective detector. They were equipped with a gas chromatography-flame ionization detector (GC-FID) and an HP-5MS (30 m × 0.25 mm × 0.25 μm) capillary column. The essential oil samples were diluted in *n*-hexane to obtain a 1% solution. The injector temperature was maintained at 250 °C. The volume injected was 1 μL. The flow rate of carrier gas (helium) was 1.0 mL/min with the mass spectrum spectra scanned from 50 to 550 *m*/*z*.

The retention indices (RI) were determined from gas chromatograms using a series of *n*-alkanes (C_5_–C_36_) under the same operating conditions. Based on RI, the chemical constituents were identified by comparing with *n*-alkanes as reference. The components of oil were identified by matching their mass spectra with computer libraries, namely Wiley 275 libraries, NIST 05, and RI from other literatures [47].

### 4.4. Fumigant Toxicity

The individual compounds were obtained from TCI Shanghai Development Co., Ltd. (Shanghai, China) (D-camphor, 1,8-cineole) and Sigma-Aldrich Shanghai Trading Co., Ltd. (Shanghai, China) (linalool and α-terpineol). The appropriate testing concentrations were determined by range-finding studies. A serial dilution of the essential oils and compounds with five concentrations was prepared in *n*-hexane. The essential oils and above samples were tested with the method described by Liu and Ho [22]. Ten insects were put inside a glass vial (diameter 2.5 cm, height 5.5 cm, volume 25 mL). On the bottom of the cap, a filter paper (diameter 2 cm) was placed and treated with a 10 µL sample solution. Before placing the cap tightly on the glass vial to form a sealed chamber, the solvent evaporated for 20 s. The negative control was *n*-hexane. The experiments were performed with five replicates for each treatment. The packets were incubated for 24 h. After that, dead insects were counted, and SPSS V20.0 Probit analysis was used to calculate LC_50_ values [48].

### 4.5. Contact Toxicity

The essential oils and individual compounds against the two stored-product insects were measured as described by Liu and Ho [22]. Range-finding studies were run to determine the appropriate testing concentrations. A series of testing samples (five concentrations) was diluted using *n*-hexane for essential oils and individual compounds. The dorsal thorax of each insect was applied to 0.5 µL aliquots of the dilution. The control group was treated only with *n*-hexane. Ten treated insects were then transferred to each glass vial and kept in the dark incubator. Each experiment was replicated five times. After 24 h, mortality was recorded, and the LD_50_ values were calculated using SPSS V20.0 Probit analysis [48].

## 5. Conclusions

The significantly high efficacy of the essential oils of *C. camphora* and its individual constituents against insects in stored products demonstrated in this study may lead to the development of new natural products. Additionally, further research could concentrate more on the safety, potency, and stability of the essential oils.

## Figures and Tables

**Table 1:** Constituents identified from the essential oils of barks (EB), leaves (EL), and fruits (EF) of *Cinnamomum camphora*.

No.	RI ^1^	Compounds	Peak Area (%)			Identified Method ^3^
			EB	EL ^2^	EF	
1	927	Artemesia triene	1.0			MS; RI
2	939	α-Pinene		2.1		MS; RI
3	952	Camphene		1.0	0.2	MS; RI
4	967	2-Thujene		2.0	0.2	MS; RI
5	977	Sabenene		1.8		MS; RI
6	979	β-Pinene	0.3			MS; RI
7	1005	α-Phellandrene		0.4	2.6	MS; RI
8	1011	*p*-Mentha-2,4(8)-diene		0.4	0.3	MS; RI
9	1014	3-Carene			0.5	MS; RI
10	1018	4-Carene	0.2			MS; RI
11	1022	*o*-Cymene			2.7	MS; RI
12	1025	*m*-Cymene		0.4		MS; RI
13	1032	1,8-Cineole	4.3	11.3	5.3	MS; RI
14	1051	α-*trans*-Ocimene		0.1	0.2	MS; RI
15	1055	2,2-Dimethylheptane		0.1		MS; RI
16	1056	γ-Terpinen	0.3			MS; RI
17	1057	2,2,5-Trimethylhexane-3,4-dione		0.1		MS; RI
18	1061	4,7-Dimethyl-4,4a,5,6-tetrahydrocyclopenta[c]pyran-1,3-dione		0.3		MS; RI
19	1067	2,5,9-Trimethyldecane		0.1		MS; RI
20	1075	Isoterpinolene	0.4			MS; RI
21	1083	Linalool		22.9	12.8	MS; RI
22	1096	Undecane	0.2			MS; RI
23	1104	1-Methyl-5-(1-methylvinyl)cyclohexene	1.6			MS
24	1108	1,3,8-p-Menthatriene	1.1			MS; RI
25	1130	7,7-Dimethyl-2-methylenenorbornane	0.5	0.1	0.3	MS; RI
26	1146	D-Camphor	51.3	40.5	28.1	MS; RI
27	1180	Terpinen-4-ol	2.0			MS; RI
28	1182	endo-Borneol		0.2		MS; RI
29	1193	α-Terpineol	3.8			MS; RI
30	1197	*p*-Menth-1-en-4-ol		1.1	0.7	MS; RI
31	1214	*p*-Menth-1-en-8-ol		2.3	1.7	MS; RI
32	1281	B-Terpinyl acetate			1.3	MS; RI
33	1287	Safrole			29.0	MS; RI
34	1354	Elixene		0.3		MS; RI
35	1356	Eugenol	2.1			MS; RI
36	1379	Dihydro-*cis*-α-copaene-8-ol	1.4	0.6	0.4	MS; RI
37	1382	α-Bourbonene	0.2	0.1		MS; RI
38	1401	1,5-Dimethyl-8-isopropenyl-1,5-cyclodeca-diene		0.2		MS; RI
39	1420	Caryophyllene		2.2		MS; RI
40	1435	Bergamotene	0.3			MS; RI
41	1439	Aromadendrene	0.9			MS; RI
42	1441	γ-Patchoulene	0.3		0.2	MS; RI
43	1458	γ-Elemene		1.0		MS; RI
44	1466	α-Cubebene	1.3			MS; RI
45	1474	Germacrene D		0.9		MS; RI
46	1478	α-Caryophyllene	0.5	0.2		MS; RI
47	1485	β-Selinene	0.4			MS; RI
48	1489	1,2,3,4,6,8alpha-Hexahydro-1-isopropyl-4,7-dimethylnaphthalene	0.2			MS; RI
49	1520	β-Cadinene	2.0			MS; RI
50	1543	α-Calacorene	0.2			MS; RI
51	1578	3,5-Dimethyl-4-octanone		0.1		MS; RI
52	1596	Cadina-1(10),4-diene		0.1		MS; RI
53	1634	3,7,11-Trimethyl-3-hydroxy-6,10-dodecadien-1-yl acetate		4.5		MS; RI
54	1672	Oxalic acid,di(1-menthyl) ester		0.4		MS; RI
55	1691	1,3,3-Trimethyl-2-hydroxymethyl-3,3-dimethyl-4-(3-methylbut-2-enyl)-cyclohexene	2.8	0.1		MS; RI
56	1799	3-Methyl-2-butenoic acid, oct-3-en-2-yl ester	3.1			MS
		Total	82.7	97.9	86.5	

^1^ Retention index (RI) relative to the homologous series of *n*-hexane on the HP-5 MS capillary column; ^2^ The results in our previous work [18]; ^3^ MS = mass spectrum.

**Table 2:** Fumigant toxicity of essential oils and individual compounds against *T*. *castaneum* (TC) and *L. serricorne* (LS) adults at 24 h.

Insects	Samples ^5^	LC_50_ (mg/L Air)	95% FL	Slope ± SE	Chi-Square	*df*
TC	EB	<3.2 (mortality 100% ± 0%)	-	-	-	23
	EL	<3.1 (mortality 100% ± 0%)	-	-	-	23
	EF	8.5	7.8–9.5	9.2 ± 0.1	12.9	23
	D-Camphor	<2.3 (mortality 87% ± 5%)	-	-	-	23
	Linalool	12.7	11.6–13.9	5.1± 0.5	18.4	23
	Safrole ^1^	38.3	35.1–41.6	-	14.9	-
	1,8-Cineole	5.5	4.7–6.2	4.0 ± 0.5	25.3	23
	α-Terpineol	>172.4	-	-	-	23
	MeBr ^2^	1.7	-	-	-	-
LS	EB	3.0	2.7–3.4	2.5 ± 0.4	5.5	23
	EL ^3^	2.5	2.2–2.9	3.6 ± 0.4	14.4	23
	EF	<3.3 (mortality 100% ± 0%)	-	–	-	23
	D-Camphor ^3^	2.4	1.9–2.7	2.7 ± 0.3	14.3	23
	Linalool ^3^	18.0	12.3–22.7	1.8 ± 0.4	16.3	23
	Safrole	-	-	-	-	-
	1,8-Cineole	5.2	4.6–5.7	4.0 ± 0.5	16.7	23
	α-Terpineol	3.3	3.2–3.4	12.1 ± 1.5	19.1	23
	Phosphine ^4^	9.23 × 10^−3^	7.13 × 10^−3^–11.37 × 10^−3^	2.1 ± 0.3	12.0	23

^1^ Date from [21]; ^2^ Date from [22]; ^3^ Date from [18]; ^4^ Date from [23]; ^5^ The mortality of the negative control was 0 for the two insects.

**Table 3:** Contact toxicity of essential oils and individual compounds against *T*. *castaneum* (TC) and *L. serricorne* (LS) adults at 24 h.

Insects	Samples ^5^	LD_50_ (µg/Adult)	95% FL	Slope ± SE	Chi-Square	*df*
TC	EB	>50.0 (mortality 28% ± 8%)	-	-	-	23
	EL	>50.0 (mortality 38% ± 8%)	-	-	-	23
	EF	19.0	17.4–20.8	5.5 ± 0.7	11.3	23
	D-Camphor	>50.0 (mortality 24% ± 5%)	-	-	-	23
	Linalool	37.3	31.9–42.3	3.3 ± 0.4	13.6	23
	Safrole ^1^	4.7	4.0-5.2	-	10.4	-
	1,8-Cineole	18.8	17.1–20.7	5.0 ± 0.5	16.6	23
	α-Terpineol	>50.0 (mortality 26% ± 5%)	-	-	-	23
	Pyrethrins ^2^	0.3	0.2–0.3	-	13.1	23
LS	EB	7.6	4.5–9.7	2.5 ± 0.4	6.5	23
	EL ^3^	21.3	19.1–23.6	4.2 ± 0.4	11.3	23
	EF	10.1	7.2–12.4	2.5 ± 0.4	6.7	23
	D-Camphor	13.4	10.4–16.1	1.5 ± 0.3	15.4	23
	Linalool	12.7	11.3–14.2	4.9 ± 0.6	13.1	23
	Safrole ^4^	14.6	12.3–16.9	2.4 ± 0.3	12.9	23
	1,8-Cineole	15.6	12.9–18.0	3.0 ± 0.6	15.2	23
	α-Terpineol	12.0	10.4–13.4	3.1 ± 0.4	19.0	23
	Pyrethrins ^2^	0.2	0.2–0.4	1.3 ± 0.2	17.4	23

^1^ Date from [24]; ^2^ Date from [23]; ^3^ Date from [18]; ^4^ Date from [21]; ^5^ The mortality of the negative control was 0 for the two insects.
